# Supplementary material for: HIVprotI: an integrated web based platform for prediction and design of HIV proteins inhibitors
Source: J Cheminform. 2018 Mar 9;10:12. doi: 10.1186/s13321-018-0266-y (PMC5845081; doi:10.1186/s13321-018-0266-y)
Supplement: Supplementary file 2 — Additional file 2. Source code of HIVProtI web server. [file 13321_2018_266_MOESM2_ESM.zip › HIVprotI_Source-code/hivprotI/weka-3-6-6/doc/weka/core/xml/PropertyHandler.html]

PropertyHandler


---


|  |  |  |
| --- | --- | --- |
| **Overview** \| **Package** \| **Class** \| **Tree** \| **Deprecated** \| **Index** \| **Help** \| **Weka's home** | |  |
| **PREV CLASS**   **NEXT CLASS** | **FRAMES**    **NO FRAMES**     **All Classes** |  |
| SUMMARY: NESTED \| FIELD \| CONSTR \| METHOD | DETAIL: FIELD \| CONSTR \| METHOD |  |


---


## weka.core.xml Class PropertyHandler

```
java.lang.Object
  weka.core.xml.PropertyHandler
```

**All Implemented Interfaces:**: RevisionHandler

---

``` public class PropertyHandler extends java.lang.Object implements RevisionHandler ```

This class stores information about properties to ignore or properties
that are allowed for a certain class.

**Version:**
:   $Revision: 1.4 $

**Author:**
:   FracPete (fracpete at waikato dot ac dot nz)

---

**Constructor Summary**

`PropertyHandler()`
  
          initializes the handling


| **Method Summary** | |
| --- | --- |
| `void` | `addAllowed(java.lang.Class c, java.lang.String displayName)`             adds the given property (display name) to the list of allowed properties for the specified class. |
| `void` | `addIgnored(java.lang.Class c, java.lang.String displayName)`             adds the given class with the display name of a property to the ignore list. |
| `void` | `addIgnored(java.lang.String displayName)`             adds the given display name of a property to the ignore list. |
| `java.util.Enumeration` | `allowed()`             returns an enumeration of the classnames for which only certain properties (display names) are allowed |
| `java.lang.String` | `getRevision()`             Returns the revision string. |
| `java.util.Enumeration` | `ignored()`             returns an enumeration of the stored display names and classes of properties to ignore.  **NOTE:** String and Class Objects are mixed in this enumeration, depending whether it is a global property to ignore or just one for a certain class! |
| `boolean` | `isAllowed(java.lang.Class c, java.lang.String displayName)`             returns whether the given property (display name) is allowed for the given class. |
| `boolean` | `isAllowed(java.lang.Object o, java.lang.String displayName)`             returns whether the given property (display name) is allowed for the given object . |
| `boolean` | `isIgnored(java.lang.Class c, java.lang.String displayName)`             checks whether the given display name of a certain class is an ignored property. |
| `boolean` | `isIgnored(java.lang.Object o, java.lang.String displayName)`             checks whether the given display name of a given object is an ignored property. |
| `boolean` | `isIgnored(java.lang.String displayName)`             checks whether the given display name is an ignored property |
| `boolean` | `removeAllowed(java.lang.Class c, java.lang.String displayName)`             removes the given property (display name) for the specified class from the list of allowed properties. |
| `boolean` | `removeIgnored(java.lang.Class c, java.lang.String displayName)`             removes the given display name from the ignore list of the class. |
| `boolean` | `removeIgnored(java.lang.String displayName)`             removes the given display name from the ignore list. |

**Methods inherited from class java.lang.Object**

`equals, getClass, hashCode, notify, notifyAll, toString, wait, wait, wait`

**Constructor Detail**

### PropertyHandler

```
public PropertyHandler()
```

:   initializes the handling


**Method Detail**

### ignored

```
public java.util.Enumeration ignored()
```

:   returns an enumeration of the stored display names and classes of
    properties to ignore.  
    **NOTE:** String and Class Objects are mixed in this enumeration, depending
    whether it is a global property to ignore or just one for a certain class!

    :   **Returns:**: the display names and classes **See Also:**: `m_Ignored`

---


### addIgnored

```
public void addIgnored(java.lang.String displayName)
```

:   adds the given display name of a property to the ignore list. Can either
    be a complete path (e.g. `__root__.options`) or only a
    property name (e.g. `options`). In the latter case it matches
    all occurences of this display name.

    :   **Parameters:**: `displayName` - the property to ignore **See Also:**: `m_Ignored`

---


### addIgnored

```
public void addIgnored(java.lang.Class c,
                       java.lang.String displayName)
```

:   adds the given class with the display name of a property to the ignore list.
    I.e. this property is only ignored for this class.

    :   **Parameters:**: `c` - the class for which a property is to be ignored: `displayName` - the property to ignore **See Also:**: `m_Ignored`

---


### removeIgnored

```
public boolean removeIgnored(java.lang.String displayName)
```

:   removes the given display name from the ignore list. returns whether the
    removing was succesful, i.e. whether the display name was in the list.

    :   **Parameters:**: `displayName` - the property to remove from the ignore list **Returns:**: whether the ignore list contained the specified property **See Also:**: `m_Ignored`

---


### removeIgnored

```
public boolean removeIgnored(java.lang.Class c,
                             java.lang.String displayName)
```

:   removes the given display name from the ignore list of the class.
    returns whether the removing was succesful, i.e. whether the display
    name was in the list.

    :   **Parameters:**: `c` - the class to remove the property from: `displayName` - the property to remove from the ignore list **Returns:**: whether the ignore list contained the specified property **See Also:**: `m_Ignored`

---


### isIgnored

```
public boolean isIgnored(java.lang.String displayName)
```

:   checks whether the given display name is an ignored property

    :   **Parameters:**: `displayName` - the property to check whether it is on the ignore list **Returns:**: whether the property is in the ignored list **See Also:**: `m_Ignored`

---


### isIgnored

```
public boolean isIgnored(java.lang.Class c,
                         java.lang.String displayName)
```

:   checks whether the given display name of a certain class is an ignored
    property. It only checks for this certain class and no derivative classes.
    If you also want to check for derivative classes, use
    `isIgnored(Object,String)`.

    :   **Parameters:**: `c` - the class to check the property for: `displayName` - the property to check whether it is on the ignore list **Returns:**: whether the property is in the ignored list **See Also:**: `m_Ignored`, `isIgnored(Object, String)`

---


### isIgnored

```
public boolean isIgnored(java.lang.Object o,
                         java.lang.String displayName)
```

:   checks whether the given display name of a given object is an ignored
    property. The object is checked for each stored class whether it is an
    `instanceof`. If the class is not stored then it will default
    to `false`, since there are no restrictions for this class.

    :   **Parameters:**: `o` - the object to check the property for: `displayName` - the property to check whether it is on the ignore list **Returns:**: whether the property is in the ignored list **See Also:**: `m_Ignored`

---


### allowed

```
public java.util.Enumeration allowed()
```

:   returns an enumeration of the classnames for which only certain properties
    (display names) are allowed

    :   **Returns:**: the classnames with restriction to properties

---


### addAllowed

```
public void addAllowed(java.lang.Class c,
                       java.lang.String displayName)
```

:   adds the given property (display name) to the list of allowed properties
    for the specified class.

    :   **Parameters:**: `c` - the class to add a property for: `displayName` - the property to allow for the class **See Also:**: `m_Allowed`

---


### removeAllowed

```
public boolean removeAllowed(java.lang.Class c,
                             java.lang.String displayName)
```

:   removes the given property (display name) for the specified class from
    the list of allowed properties.

    :   **Parameters:**: `c` - the class to remove the property for: `displayName` - the property to remove **Returns:**: whether the property was found **See Also:**: `m_Allowed`

---


### isAllowed

```
public boolean isAllowed(java.lang.Class c,
                         java.lang.String displayName)
```

:   returns whether the given property (display name) is allowed for the
    given class. It only checks for this certain class and no derivative classes.
    If you also want to check for derivative classes, use
    `isAllowed(Object,String)`.

    :   **Parameters:**: `c` - the class to check the property for: `displayName` - the property (display name) to check **Returns:**: whether the property is allowed in that context **See Also:**: `m_Allowed`, `isAllowed(Object, String)`

---


### isAllowed

```
public boolean isAllowed(java.lang.Object o,
                         java.lang.String displayName)
```

:   returns whether the given property (display name) is allowed for the given
    object . The object is checked for each stored class whether it is an
    `instanceof`. If the class is not stored then it will default
    to `true`, since there are no restrictions for this class.

    :   **Parameters:**: `o` - the object to check the property for: `displayName` - the property (display name) to check **Returns:**: whether the property is allowed in that context

---


### getRevision

```
public java.lang.String getRevision()
```

:   Returns the revision string.

    :   **Specified by:**: `getRevision` in interface `RevisionHandler`
    :   **Returns:**: the revision


---


|  |  |  |
| --- | --- | --- |
| **Overview** \| **Package** \| **Class** \| **Tree** \| **Deprecated** \| **Index** \| **Help** \| **Weka's home** | |  |
| **PREV CLASS**   **NEXT CLASS** | **FRAMES**    **NO FRAMES**     **All Classes** |  |
| SUMMARY: NESTED \| FIELD \| CONSTR \| METHOD | DETAIL: FIELD \| CONSTR \| METHOD |  |


---
